# Optimization of Multimeric Human Papillomavirus L2 Vaccines

**DOI:** 10.1371/journal.pone.0055538

**Published:** 2013-01-31

**Authors:** Subhashini Jagu, Kihyuck Kwak, Balasubramanyam Karanam, Warner K. Huh, Vijayarangam Damotharan, Sudha V. Chivukula, Richard B. S. Roden

**Affiliations:** 1 Department of Pathology, Johns Hopkins University, Baltimore, Maryland, United States of America; 2 Department of Oncology, Johns Hopkins University, Baltimore, Maryland, United States of America; 3 Department of Gynecology and Obstetrics, Johns Hopkins University, Baltimore, Maryland, United States of America; 4 Department of Gynecologic Oncology, University of Alabama at Birmingham, Birmingham, Alabama, United States of America; 5 Shantha Biotechnics Limited, Hyderabad, Andhra Pradesh, India; National Institute of Health - National Cancer Institute, United States of America

## Abstract

We sought to define the protective epitopes within the amino terminus of human papillomavirus (HPV) type 16 minor capsid protein L2. Passive transfer of mice with rabbit antisera to HPV16 L2 peptides 17–36, 32–51 and 65–81 provided significant protection against vaginal HPV16 challenge, whereas antisera to 47–66, 108–120 or 373–392 did not. Vaccination with L1 virus-like particles induces a high titer, but generally type-restricted neutralizing antibody response. Conversely, vaccination with L2 11–88, especially multimers thereof, induces antibodies that neutralize a broad range of papillomavirus types, albeit at lower titers than for L1 VLP. With the intent of enhancing the immunogenicity and the breadth of protection by focusing the immune response to the key protective epitopes, we designed L2 fusion proteins consisting of residues ∼11–88 of eight divergent mucosal HPV types 6, 16, 18, 31, 39, 51, 56, 73 (11–88×8) or residues ∼13–47 of fifteen HPV types (13–47×15). The 11–88×8 was significantly more immunogenic than 13–47×15 in Balb/c mice regardless of the adjuvant used, suggesting the value of including the 65–81 protective epitope in the vaccine. Since the L2 47–66 peptide antiserum failed to elicit significant protection, we generated an 11–88×8 construct deleted for this region in each subunit (11–88×8Δ). Mice were vaccinated with 11–88×8 and 11–88×8Δ to determine if deletion of this non-protective epitope enhanced the neutralizing antibody response. However, 11–88×8Δ was significantly less immunogenic than 11–88×8, and even the addition of a known T helper epitope, PADRE, to the construct (11–88×8ΔPADRE) failed to recover the immunogenicity of 11–88×8 in C57BL/6 mice, suggesting that while L2 47–66 is not a critical protective or T helper epitope, it nevertheless contributes to the immunogenicity of the L2 11–88×8 multimer vaccine.

## Introduction

The efficacy of vaccination with HPV L1 virus-like particles (VLP) for the prevention of new infections provides an opportunity to reduce the incidence of HPV-associated cancers globally if these vaccines can be widely utilized [1], [2], [3], [4], [5]. This opportunity is particularly dramatic for women who currently lack access to effective cytologic screening and intervention programs. Indeed, 85% of the global burden of disease occurs in such low income countries [6]. Unfortunately, the current cost of the licensed L1 VLP vaccines has proven a significant barrier to their sustained global implementation, and this has driven an effort to create a second generation of low cost HPV vaccines that require fewer doses to improve access for under-served populations [7]. The licensed HPV vaccines target only the two types most commonly found in cervical cancer, HPV16 and HPV18 that cause 70% of cases, but there are a dozen other types responsible for remaining ∼30% of cervical cancer cases [8]. The L1 VLP vaccines provide type-restricted protection and, while a variable degree of cross-protection against highly related types has been described, there is concern that it is incomplete and may wane [5], [9]. This has triggered an ongoing clinical effort to develop a nonavalent L1 VLP vaccine, but its potential to further increase the cost of vaccination against HPV has encouraged the development of alternate vaccines based on the more cross-protective capsid antigen L2 [7].

L2 can be produced at high levels in bacteria and numerous studies demonstrate it is a protective antigen although it does not form a VLP [10], [11], [12], [13]. Vaccination of rabbits with the N-terminus (residues 94–122, 11–200 or 1–88) of L2 prevents papilloma development after experimental challenge with virions but not viral DNA, suggesting that protection is mediated by neutralizing antibodies [13], [14]. Indeed, neutralizing antibodies binding to linear epitopes in HPV16 L2 17–36, 65–81 and 108–120 have been described [15], [16], [17]. The development of HPV pseudovirion (PsV) technology in which a reporter gene is encapsidated within the papillomavirus L1 and L2 capsid has greatly facilitated the measurement of neutralizing antibodies, and recently has been utilized in a mouse challenge model [18], [19]. Passive transfer of the HPV16 L2 17–36 specific neutralizing antibody RG-1 protected naïve mice from cutaneous challenge with HPV16 PsVs suggesting that L2-specific neutralizing IgG is sufficient to mediate protection [15].

Antisera to the N-terminus of L2 broadly cross-neutralizes HPV, although it is most effective against the virus type from which the vaccine was derived, and the titers induced are significantly lower than those produced by L1 VLP vaccines [20], [21]. The induction of sustained neutralizing antibody titers for durable/lifetime protection is a critical goal and might offer an opportunity to move from an adolescent to childhood vaccination schedule to further improve vaccine access. To potentially enhance the level, durability and breadth of cross-protection by reinforcing the most conserved epitopes, we designed concatenated fusion proteins consisting of the N-terminal protective region of L2 derived from multiple medically significant HPV genotypes [22]. This study suggested that a pentameric fusion of L2 residues of 11–88 from divergent HPV types could induce a robust humoral response, but another study suggested that inclusion of more repeats might be beneficial [23]. Herein we define HPV16 L2 residues 17–36, 32–51 and 65–81 as protective epitopes, and show an unexpected enhancement of the neutralizing antibody response to the amino terminus of L2 by residues 45–67.

## Methods

### Ethics Statement

This study was carried out in strict accordance with the recommendations in the Guide for the Care and Use of Laboratory Animals of the National Institutes of Health. All animal studies were performed with the prior approval of the Animal Care and Use Committee of Johns Hopkins University (protocol MO08M19).

### Antigen preparation

The L2 multimer constructs 11–88×8, 13–47×15, 11–88×8Δ, and 11–88×8Δ PADRE were codon optimized for expression in *E. coli* by lowest free energy calculation and synthesized by Blue Heron Inc. (Methods S1). The 11–88×8 family constructs were cloned with BamHI sites at their N-terminus and XhoI sites at their C terminus into the pET28a vector (Novagen), whereas NheI and XhoI were used for 13–47×15. The N-terminal hexahistidine-tagged recombinant polypeptides were expressed in *E. coli* BL21 (Rosetta cells, Novagen) [21]. The recombinant L2 polypeptides were affinity purified by binding to a nickel-nitrilotriacetic acid (Ni-NTA) column (Qiagen) in 8 M urea (using the QiaExpressionist standard purification protocol for denaturing conditions) and then dialyzed in cassettes (Pierce) against Dulbecco's phosphate buffered saline (PBS). Purity was monitored by SDS-PAGE and protein concentration determined by bicinchoninic acid test (Pierce) using a bovine serum albumen standard. L2 peptides were synthesized with a C-terminal cysteine to >90% purity, sequence validated by mass spectrometry and 10 mg conjugated with 5 mg keyhole limpet hemocyanin (KLH) protein carrier using maleimide (Proteintech) to enhance their immunogenicity [24]. Two rabbits were immunized with each KLH-coupled peptide in Freund's adjuvant and boosted on days 14, 28, 35 and 76 using incomplete Freund's adjuvant and exsanguinated at day 56 (Proteintech).

### Neutralization assays

The HPV pseudovirion *in vitro* neutralization assays were performed as described earlier and the secreted alkaline phosphatase content in the clarified supernatant was determined using the *p*-Nitrophenyl phosphate tablets (Sigma, St. Louis, MO) dissolved in diethanolamine and absorbance measured at 405 nm. Constructs and detailed protocols for the preparation of the pseudovirions can be found at http://home.ccr.cancer.gov/lco/. Titers were defined as the reciprocal of the highest dilution that caused a 50% reduction in A_405_, and a titer <50 was not considered significant.

### Animal Studies

Female Balb/c mice, 4–6 weeks age (NCI Frederick) were vaccinated in groups of 5 animals three times at two week intervals s.c with 25 µg of antigen (11–88×8, 13–47×15, 11–88×8Δ, 11–88×8ΔPADRE) either with Alum alone, or Alum+MPL, Alum+CpG 1018 (Dynavax Inc.) or GPI-0100 (Hawaii Biotech Inc.). Serum samples were obtained by tail vein bleeds two weeks and four months after the final immunization.

### Passive transfer of mice and vaginal challenge with HPV pseudovirus

Five days before the challenge, female Balb/c mice were injected s.c. with 3 mg of medroxyprogesterone (Depo-Provera; Pfizer) to synchronize their estrus cycles. For those mice receiving the passively transferred rabbit antiserum, 100 µl of serum was administered intraperitoneally 24 hours prior to infection. Each challenge dose comprised of 20 µl PsV mixed with 20 µl of 3% carboxymethyl cellulose (CMC). The dose was delivered twice into vaginal vault, the first 20 µl just prior to and the second 20 µl just after treatment with a cytobrush cell collector. The cytobrush cell collector was inserted into the vaginal vault and turned both counter-clockwise and clockwise 15 times while the mice were anesthetized. Three days after PsV delivery, the mice were again anesthetized and 20 µl of luciferin (7.8 mg/ml) was deposited in the vaginal vault. Luciferase signals were acquired for 10 min with a Xenogen IVIS 100 imager, and analysis was performed with Living Image 2.5 software.

### Statistical analysis

One-way ANOVA and Bonferroni's multiple comparison test were performed with GraphPad 4.00 (GraphPad Software, San Diego, CA).

## Results

### Passive transfer with L2 peptide anti-sera protects mice from vaginal HPV16 challenge

**Figure 1:**
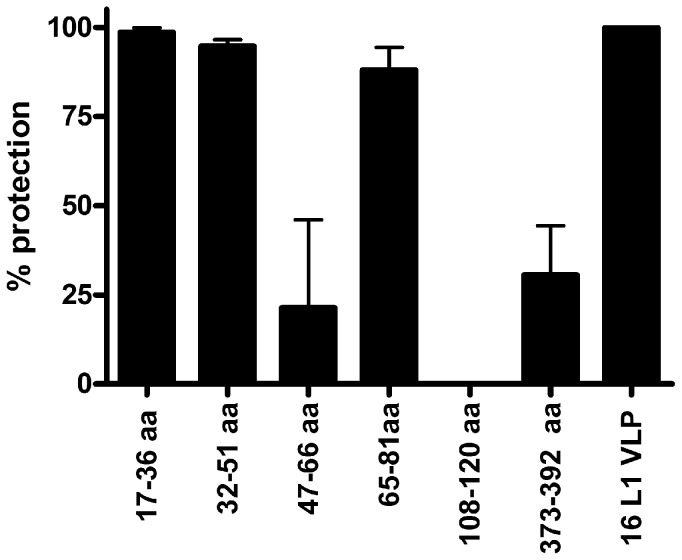
Passive transfer of HPV16 L2 peptide antisera protects mice against vaginal challenge with HPV16. Mice were injected i.p. with 0.1 ml buffer or rabbit antiserum to KLH-coupled HPV16 L2 peptides encompassing residues 17–36, residues 32–51, residues 47–66, residues 65–81, 108–120, residues 373–392, or antiserum to HPV16 L1 VLP. One day later the mice were challenged intra-vaginally with HPV16 pseudovirion encoding luciferase.

To define protective epitopes within the amino terminus of L2, rabbits were vaccinated with KLH-coupled synthetic HPV16 L2 peptides comprising residues 17–36, 32–51, 47–66, 65–81, 108–120 or as a negative control C-terminal peptide 373–392, each formulated initially in CFA and boosted in IFA. The pre- and hyper-immune antisera were each tested for reactivity with full length HPV16 L2 protein by ELISA. None of the pre-immune sera were reactive whereas the immune sera to HPV16 L2 17–36, 32–51, 47–66, 65–81, 108–120, and 373–392 exhibited ELISA titers of 6400, 400, 100, 800, 800, and 6400 respectively. Passive transfer of 0.1 ml/mouse rabbit anti-sera generated against HPV16 L2 residues 17–36, 32–51 and 65–81 protected naïve Balb/c mice (n = 5) against intra-vaginal challenge with HPV16 PsV one day later (p<0.01, p<0.05 and p<0.05 respectively, Figure 1). Conversely, passive transfer of 0.1 ml/mouse of antisera to HPV16 L2 residues 47–66 (n = 10), 108–120 (n = 5) or 373–392 (n = 5) was not significantly protective. A second rabbit immunized with the HPV16 L2 47–66 peptide did not produce a detectable titer in the HPV16 L2 protein ELISA and also was not protective (not shown), suggesting that this peptide is either poorly immunogenic and/or was not presented in the appropriate context for immunization. Passive transfer of rabbit antiserum to HPV16 L1 VLP also protected mice from vaginal challenge (n = 5, Figure 1).

### Antibody responses to different adjuvant formulations of 11–88×8 and 13–47×15

Recent studies have shown that vaccination with polymeric fusions of L2, each unit being derived from a different HPV type, produces a more broadly neutralizing response than the monomer, and that increasing the numbers of the fusion epitopes can enhance immunogenicity [22], [23]. To potentially enhance cross-protection by reinforcing the responses to conserved neutralizing epitopes within the medically significant α7, α9 and α10 clades [25], we generated two multitype L2 fusion proteins, 11–88×8 and 13–47×15, by recombinant expression in *E. coli* [22]. The first, 11–88×8, comprised L2 residues ∼11–88 of HPV6, HPV16, HPV18, HPV31, HPV39, HPV51, HPV56 and HPV73 L2 concatenated to form the ‘11–88×8’ antigen, and the second, 13–45×15, comprised L2 residues ∼13–47 of the fifteen HPV types (HPV6, HPV11, HPV 16, HPV18, HPV31, HPV33, HPV35, HPV39, HPV45, HPV51, HPV52, HPV56, HPV58, HPV59, HPV73) that encompass the two most common types in genital warts and the thirteen most common oncogenic HPV types. Both proteins were expressed in bacteria and affinity purified under denaturing conditions utilizing an N-terminal 6His tag. The expression level of 13–45×15 was noticeably higher than 11–88×8.

**Figure 2:**
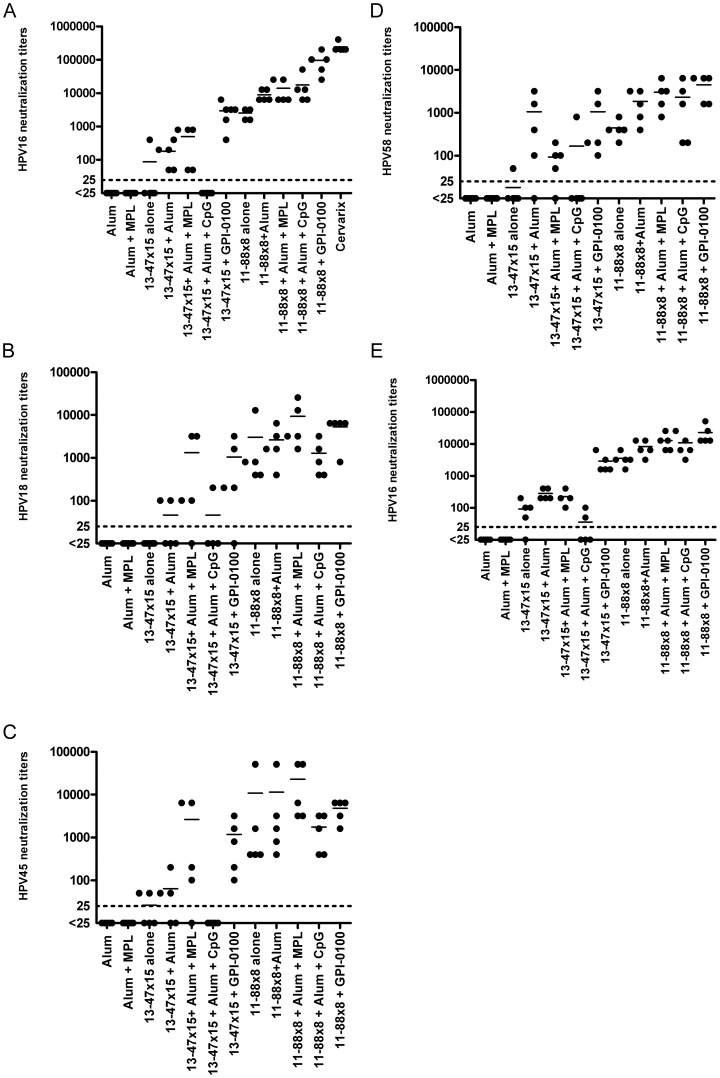
HPV in vitro neutralization titers of sera of mice vaccinated with 13–47×15 and 11–88×8 in different adjuvants. Balb/c mice were vaccinated three times at 2 week intervals with the indicated 13–47×15 and 11–88×8 proteins formulated in alum, alum+MPL, alum+CpG or GPI-0100. Sera were harvested two weeks later for testing in vitro neutralization titers against HPV16 (A), HPV18 (B), HPV45 (C) and HPV58 (D) pseudovirions, or HPV16 at 3 months after the final vaccination (E).

In order to compare the immunogenicity of the 11–88×8 and 13–45×15 antigens, mice were immunized three times at two week intervals with either 13–47×15 or 11–88×8 protein, alone or formulated with alum alone, alum+MPL, alum+CpG 1018, or the saponin-based adjuvant GPI-0100. Both the 11–88×8 and 13–45×15 polypeptides were immunogenic and induced antibodies that neutralize key papillomavirus types, including HPV16, HPV18, HPV45 and HPV58 (Figure 2). However, the 13–47×15 protein was not as immunogenic as 11–88×8, regardless of the adjuvant used. Amongst the adjuvants tested, GPI-0100 was the most effective, producing titers a log higher than alum and either MPL or CpG. The HPV16 neutralization titers at 3 months post vaccination were robust and similar to those measured at 2 weeks post immunization for 11–88×8, indicating that the titers were stable, whereas the response to 13–45×15 remained weak.

### Elimination of residues 45–67 compromises 11–88×8 immunogenicity

These findings suggested that residues 45–88 contribute significantly to the immunogenicity of the construct, consistent with the protective epitope described between residues 65–81 (Figure 1). However, residues 47–66 do not appear to be immunogenic and antisera to this peptide was not significantly protective (Figure 1), suggesting that it might be dispensable from the vaccine construct. Furthermore, while the 13–47×15 protein was highly expressed, the 11–88×8 protein was produced at a significantly lower level (data not shown) possibly reflecting the hydrophobicity of L2 between residues 45–67 (ILQYGSMGVFFGGLGIGTGSGTG). In an effort to focus the antibody response on the key protective epitopes by removing potentially competing non-protective regions, we designed 11–88×8Δ by removing this region from each unit of the 11–88×8 protein. The expression level of 11–88×8Δ was dramatically higher than 11–88×8 and, unlike the 13–45×15, it still contains the neutralizing epitopes residing with residues 65–81.

While CD4 T cell epitopes are found within E2, E6, E7 and L1 and at this point it is unknown where within the L2 capsid protein a potential epitope would be located, if any, we postulated the existence of a key T helper epitope might possibly overlap or lie within the 45–67 region as a reason for the greater immunogenicity of the 11–88×8 as compared with the 11–88×8Δ polypeptide. Interestingly, an *in silico* analysis of the 11–88×8 sequence using ProPred predicts that the 45–67 region contains promiscuous MHC class II epitopes [26], [27], [28]. Thus, in removing this 45–67 region from each unit of 11–88×8, we were concerned that this would also eliminate potentially important epitopes recognized by CD4 T helper cells. Therefore, we generated another construct in which the potent and conserved CD4 T helper epitope PADRE (AKFVAAWTLKAAA) was fused to the 11–88×8Δ protein, forming ‘11–88×8ΔPADRE’. The PADRE epitope was chosen because it is recognized in C57BL/6 mice, but not Balb/c mice, and it is broadly recognized by human HLA-DR [29], [30]. Both the 11–88×8Δ protein and the 11–88×8ΔPADRE proteins were expressed in *E. coli* at noticeably higher levels than 11–88×8.

**Figure 3:**
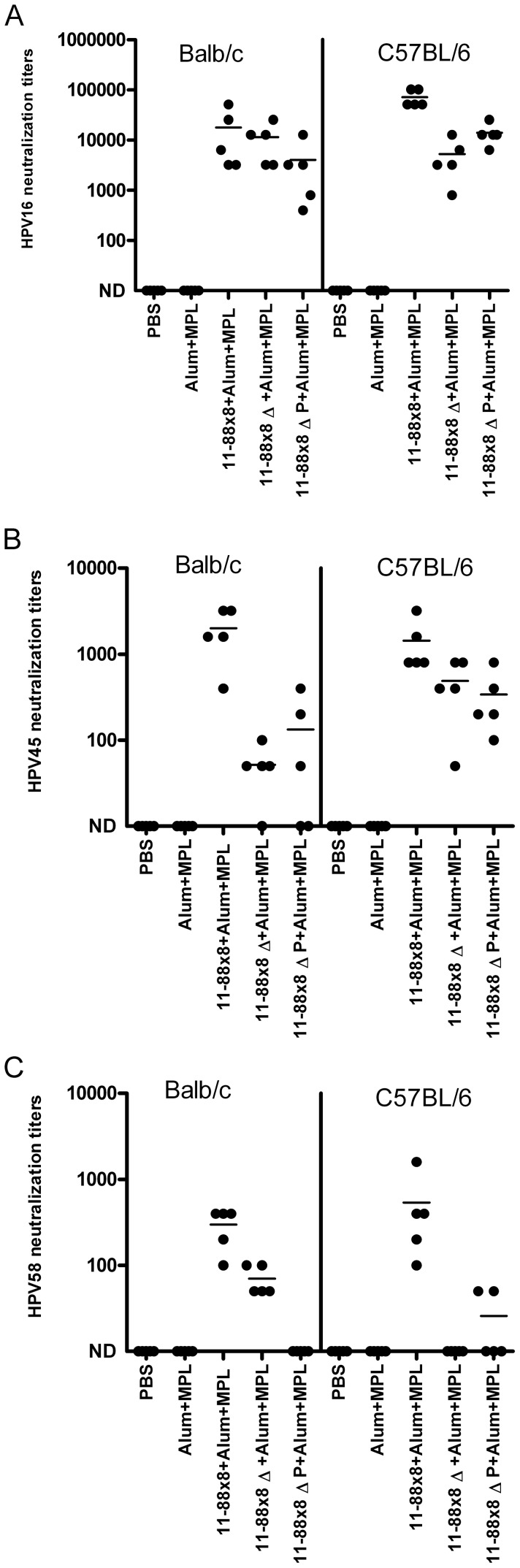
HPV in vitro neutralization titers of sera of Balb/c and C57BL/6 mice vaccinated with 11–88×8, 11–88×8Δ and 11–88×8ΔPADRE using Alum+MPL as adjuvant. Balb/c or C57BL/6 mice were vaccinated three times at 2 week intervals with 11–88×8, 11–88×8Δ or 11–88×8ΔPADRE (11–88×8ΔP) using Alum+MPL as adjuvant. Sera were harvested two weeks later and tested for in vitro neutralization titers against HPV16 (A), HPV45 (B) and HPV58 (C) pseudovirions.

**Figure 4:**
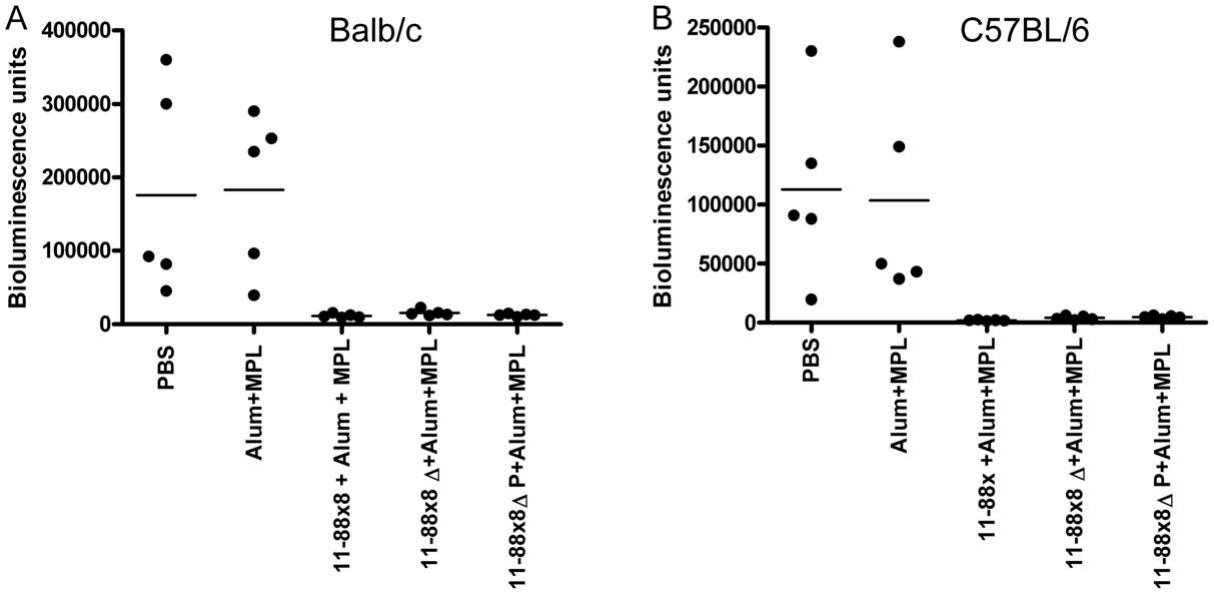
Vaginal Challenge of Balb/c and C57BL/6 mice vaccinated with 11–88×8, 11–88×8Δ and 11–88×8ΔPADRE (11–88×8ΔP) in Alum+MPL. Balb/c or C57BL/6 mice were vaccinated three times at 2 week intervals with the indicated 11–88×8, 11–88×8Δ and 11–88×8ΔPADRE using Alum+MPL as adjuvant. The mice were vaginally challenged one month later using HPV16 pseudovirions carrying a luciferase reporter. Infection is measured as bioluminescence.

To determine the impact of deleting the 45–67 region upon the immunogenicity of 11–88×8, immunization studies were performed in Balb/c and C57BL/6 mice with 11–88×8, 11–88×8Δ, or 11–88×8ΔPADRE using Alum+MPL as an adjuvant. The mice were immunized three times at two week intervals and sera were obtained two weeks after the final immunization. Neutralization assays for HPV16, HPV45, and HPV58 indicated that the titers were consistently lower in the 11–88×8Δ and 11–88×8ΔPADRE immunized mice as compared to those vaccinated with 11–88×8. This phenomenon was observed in both strains of mice (Figure 3) while the PADRE epitope is only recognized by the C57BL/6 mice. This suggests that the reduction in immunogenicity does not reflect a loss of a key T helper epitope upon elimination of the 45–67 region in each subunit of 11–88×8. Nevertheless, all three immunogens were able to protect both strains of mice from vaginal challenge with HPV16 pseudovirions (Figure 4).

### Antibody to residues 45–67 is not required for protection by 11–88×8 antiserum

**Figure 5:**
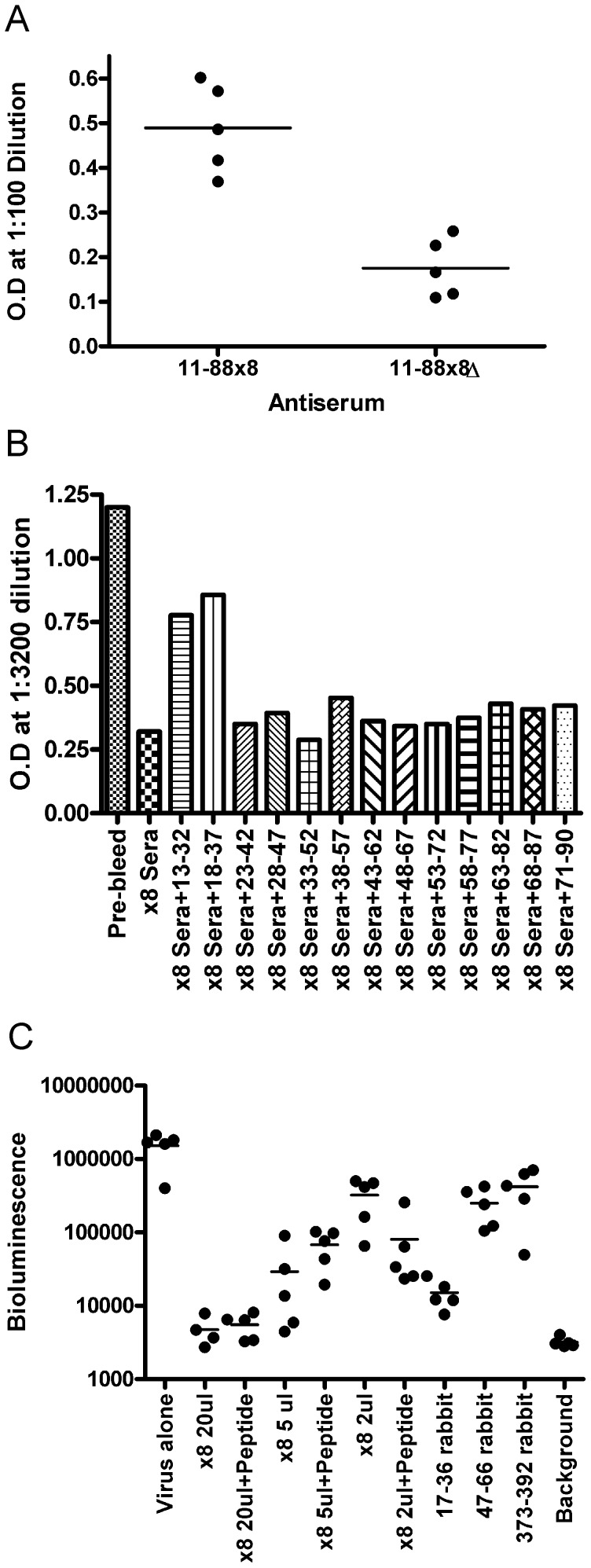
Peptide blockade of 11–88×8 antiserum. A. Sera of Balb/c mice immunized three times with 11–88×8 or 11–88×8Δ using Alum+MPL as adjuvant were harvested two weeks after the final boost. The antisera diluted 1∶100 were reacted with microtiter plates coated with 1 µg of two HPV16 L2 peptides encompassing residues 43–62 and 48–67. After washing, specific reactivity was measured by ELISA using peroxidase-linked anti-mouse IgG. B. Pooled sera of Balb/c mice immunized three times with 11–88×8 using Alum+MPL as adjuvant were harvested either pre-immunization (Pre-bleed) or two weeks after the final boost (×8 Sera). The antiserum to 11–88×8 was diluted 1∶50 in a total volume of 200 µl and incubated with 7 µg of HPV16 L2 peptide encompassing residues 13–32, 18–37, 23–42, 28–47, 33–52, 38–57, 43–62, 48–67, 53–72, 58–77, 63–82 or 71–90 for an hour prior to mixing with HPV16 pseudovirions carrying a SEAP reporter for a further hour at ambient temperature and subsequent infection of 293TT cells. Infection was measured as optical density, and only HPV16 L2 pepitdes 13–32 and 18–37 substantially blocked neutralization by 1∶3200 dilution of antiserum to 11–88×8. C. Sera of Balb/c mice immunized three times with 11–88×8 using Alum+MPL as adjuvant were harvested two weeks after the final boost and pooled (×8). The 11–88×8 antiserum was mixed with two HPV16 L2 peptides encompassing residues 43–62 and 48–67 (+Peptide) in the same ratios as in B. The 11–88×8 antiserum was administered i.p. either alone or pre-mixed with peptide in volumes of 20 µl, 5 µl or 2 µl to naïve mice (in groups of 5). Separate groups of mice received 200 µg each of antibody affinity purified with protein G columns from the sera of rabbits hyper-immunized with HPV16 L2 peptides 17–36, 47–66 or 373–392. All groups of mice (except the Background group) were subsequently challenged intra-vaginally with HPV16 pseudovirions carrying a luciferase reporter. Infection was assessed by measuring bioluminescence three days later.

To determine if antibody to residues 45–67 plays a role in the protective response elicited by vaccination with 11–88×8, we first sought to confirm that pooled mouse antisera to 11–88×8 does contain antibody specific to the 43–67 region. Using ELISA, we observed that vaccination with 11–88×8 does induce antibody reactive with 43–67 peptide at 1∶100 dilution, whereas the 11–88×8Δ antigen does not (as expected since this epitope is deleted from this construct) as it exhibits background reactivity (Figure 5A). Next, we sought to define which epitopes are critical by blocking neutralization of HPV16 pseudovirus by 11–88×8 antisera with overlapping peptides (20mer) that together span the entire HPV16 L2 13–90 region. We observe that only mixing of the 11–88×8 antisera with excess of the first two peptides, corresponding to residues 13–32 and 18–37, block the neutralizing response, whereas peptide 23–42 and peptides thereafter had minimal impact on in vitro neutralization. This again supports the central role of the 17–36 region in neutralization and protection. To further rule out a role for the 47–66 region in protection, we mixed the 11–88×8 antiserum with excess peptides encompassing the 47–66 region and compared its protective capacity against HPV16 challenge. Thus 20 µl, 5 µl or 2 µl of 11–88×8 mouse antiserum was mixed with excess 47–66 peptide, or not, and then administered i.p to naïve mice (5 mice/group) prior to challenge. As controls, additional groups of mice received 200 µg of antibody purified from the rabbit antisera to HPV16 L2 17–36, 47–66 or 373–392 (approximately 20-fold higher doses than utilized in Figure 1). The pre-incubation of the 47–66 peptide with the 11–88×8 mouse antiserum had no significant impact upon its protective capacity in this model, further supporting the notion that antibodies to 47–66 are not effecting protection after vaccination of mice with 11–88×8. Furthermore, the antibody to 17–36 but not that to 47–66 or 373–392, was strongly protective.

## Discussion

Vaccination studies in several challenge models indicate that the amino terminus of L2 has potential as a protective antigen [10]. There are ongoing efforts to map potentially protective epitopes by the generation of antisera to synthetic L2 peptides or the generation of monoclonal antibodies and testing of their *in vitro* neutralizing activity [15], [16], [17], [21], [31], [32]. We extend these studies by showing that passive transfer of antisera to HPV16 L2 residues 17–36, 32–51, and 65–81 is protective, whereas an antiserum to HPV16 L2 108–120 was not protective. These findings are generally consistent with prior in vitro neutralization studies, with the exception of the antiserum to HPV16 L2 108–120 [32], [33], [34]. However, we note that Rubio et al showed that both neutralizing and non-neutralizing antibodies can bind to the HPV16 L2 18–38 epitope, and thus it is possible that the HPV16 L2 108–120 peptide coupled to KLH utilized here for immunization induced only non-neutralizing antibodies despite containing a neutralizing epitope. Alternatively, the avidity of the neutralizing antibodies induced in the rabbit antiserum to HPV16 L2 108–120 peptide coupled to KLH was insufficient to afford protection in vivo at the dilution used (100 ul of serum in ∼2 ml plasma volume of a mouse suggests ∼1∶20). We favor the latter possibility since we previously showed that this antiserum to HPV16 L2 108–120 peptide at 1∶50 can partially neutralize native HPV11 virions [35].

Vaccination with the 11–88×8 protein induced a robust and broadly neutralizing antibody response (11–88×8 antiserum neutralizes eleven HPV genotypes, Kwak K et al, International Papillomavirus Meeting, Montreal, Canada, July 2010, Abstract P-201, http://hpv2010.org/main/images/stories/hpv2010_abstracts.pdf) and protected mice from vaginal challenge with HPV16. Surprisingly, the 13–47×15 was significantly less immunogenic than 11–88×8, and this likely reflects in part the absence of the second neutralizing epitope within residues 65–81. Similarly, we previously observed that 17–36×22 was less immunogenic than 11–88×5 or 11–200×3 [22]. However, 11–88×8Δ (from which the 45–67 equivalent region has been eliminated from each unit of L2) was also less immunogenic than 11–88×8. Since passive transfer of mice with rabbit HPV16 L2 47–66 antiserum was not significantly protective against HPV16 challenge and the peptide was poorly immunogenic, this implies that 11–88×8Δ is not less immunogenic than 11–88×8 because a potent protective epitope within the 45–67 region was deleted. However, a recent study defined the epitope for a neutralizing monoclonal antibody (Mab24B) between residues 58–64 [36]. Since the Mab24B antibody was generated in Balb/c mice, and our failure to detect neutralizing activity in the L2 47–66 peptide antiserum likely reflects its low immunogenicity in rabbits. Nevertheless, when we performed blockade studies by mixing individual overlapping 20mer peptides encompassing the N-terminus of HPV16 L2 with the mouse antiserum to 11–88×8, only peptides encompassing residues 13–32 and 18–37 impacted the neutralizing activity, suggesting the protective response is focused on this region in mice.

An *in silico* analysis using ProPred suggested that the reduction of immunogenicity by elimination of the 45–67 region in each unit of L2 within 11–88×8 might reflect the loss of CD4 T helper epitope(s). The C57BL/6 mouse strain is known to generate strong helper T cell response to PADRE, whereas Balb/c mice do not. Therefore a PADRE epitope was included in the 11–88×8ΔPADRE protein to determine if it could complement the loss of T helper epitope(s) in the 45–67 region and restore the immunogicity to that of 11–88×8 in C57BL/6 mice [30]. Since the 11–88×8ΔPADRE and 11–88×8Δ were both similarly less immunogenic than the 11–88×8 in either C57BL/6 or Balb/c mice, this suggests that loss of critical T help is not responsible for their lower immunogenicity than 11–88×8.

Although we note that the L2 multimers are purified under denaturing conditions and evidence to date suggests that the L2 neutralizing epitopes are linear, another possibility is that the 45–67 region within each unit of L2 contributes to the immunogenicity of 11–88×8 by maintaining the appropriate spacing, structure or conformation of L2 neutralizing epitopes. Thus, while the immunogens are initially purified under denaturing conditions in urea, we speculate that the 45–67 region may facilitate the folding or association of the L2 multimers under more physiologic conditions (as the urea is removed during dialysis) into a structure that better presents the neutralizing epitopes to the immune system. Indeed, recent work by Bronnimann *et al* suggests a structural role for the GxxxG motifs in this 45–67 region [37].

The observations herein support the use of the ∼11–88 region of L2 as an appropriate subunit of a multimeric L2 vaccine, but do not fully address the optimal number of subunits to include within the construct. Prior studies by Rubio with repeated units of HPV16 L2 epitopes and our own work with repeated L2 units derived from different HPV types, suggest that more than 3 subunits is optimal, and that there is little difference between 5–9 subunits and that greater numbers of repeats are not necessary, and possibly detrimental [22], [23]. Indeed, the 13–45×15 was less immunogenic than 11–88×8Δ, but it is not clear whether it reflects too many subunits, perhaps too closely spaced (17–36×22 was also poorly immunogenic), or the loss of a key neutralization epitope between residues 65–81 [16]. An alternative approach to enhance immunogenicity is to display these protective L2 epitopes on the surface of virus-like particles in immunodominant locations [38], [39]. While this complicates the use of sequences derived from multiple HPV types to enhance the breadth of cross-protective responses, virus display may enhance the strength and duration of the immune response to appropriately presented sequences, and several groups are pursuing this strategy [40], [41], [42], [43].

## Supporting Information

Methods S1 **Vaccine constructs inserted into pET28a.**
(DOCX)
