# Supplementary material for: Optimization of Multimeric Human Papillomavirus L2 Vaccines
Source: PLoS One. 2013 Jan 31;8(1):e55538. doi: 10.1371/journal.pone.0055538 (PMC3561222; doi:10.1371/journal.pone.0055538)
Supplement: Methods S1 — Vaccine constructs inserted into pET28a. (DOCX) [file pone.0055538.s001.docx]

**Multimeric L2 Construct Sequences (for supplemental data)**

**11-88x8 Sequence**

ggatccatggcctcagcaacacaactgtaccaaacttgcaaactgactggcacttgcccgccggacgttattccgaaagtagaacacaacacgatcgcggaccaaatcctgaaatggggtagtttaggcgtattttttggaggtttaggcatcggcacaggcagtggcaccggcggccgcacaggttacgtaccacttcagacctcagcgaaaccctctatcacctcaggtccaatggcaaaacgcgcaagtgcaacccagttatacaaaacttgcaaacaggcaggcacttgtccaccagacattatccctaaagtagaaggcaaaaccatcgcggatcagatcctgcaatacggctcaatgggcgtctttttcggcggtttaggcattggcactggttctggtactgggggccgcaccggctatattccgttgggcacccgtcctccaacggctacagataccctcgcccctcgtgcatcagtcaccgacctttataaaacttgtaaacagagcggcacctgtccacccgacgttgtccctaaagttgaaggcaccaccttggctgataaaattctgcaatggtcttcgctcggaatcttcttaggcggcttaggcatcggaaccggatcgggaacgggcggacgtactggttacattccactgggtggacgttctaacaccgttgttgatgtcggcccgacacgcaaacgtgcatcagccacccaactgtaccaaacttgcaaagcggctgggacttgcccgtcagacgtcatcccgaaaattgaacacacgaccattgctgaccaaatcctgcgttatggatctatgggagtcttcttcggcggtctcggtatcggttccggctctggtaccggcgggcgcacaggttatgtaccactgagtacgcgtccatcaactgtttcggaagcatcgattccgcgtgctagcgctacagatttataccgcacgtgtaaacaatctggcacatgtcccccagatgtggtcgacaaagtggaaggtacgaccctggcagacaaaatcctgcagtggacctctttaggcattttccttggtggcctcggtattggcacgggtacaggcactggtggtcgcacaggctatattcctcttggtggtcgtccgaatacggttgttgatgtatcccccgctcgtcgtgcgtcggttacccagttatattctacctgcaaagcggccggcacctgtccccctgatgttgtgaataaagttgaaggtacaaccctggccgataaaattctgcaatggagcggtttaggcatttttttaggtggccttggcatcggaacaggcagtggatctggcggtcgtacgggctacattcctttaggtggtggcggccgccctggtgtcgtagatatcgcccccgcgcgggccagcgctacgcaactgtacaaaacctgcaaactgagcggtacctgtcctgaagatgttgttaacaaaattgaacaaaagacttgggctgataaaattttacagtggggcagtttgttcacctatttcggcggactgggtatcggcactggcacaggttctggcggccgtgcaggctacgtacccttaggctcacgtccttctaccatcgttgacgttactccagcacggaaaaaacgcgcctcggcgacgcagctgtataaaacttgcaaacaggctggcacttgcccaccagatgttatcccaaaagtcgaaggtagcaccatcgctgacaacattttaaaatatgggtccattggagtatttttcggaggcctgggaatcggcagcggttctggttcaggtggccgtacaggatacgttcctctgagcaccggcaccccgtccaaacccgttgaaattccttaactcgag

**13-47x15 Sequence**

tctagacaccatggctagcgcaacccaactgtaccaaacttgcaagctgactggcacttgcccaccggacgtgatcccgaaggtggaacataacaccatcgctgatcaaatcctgaaggcgtctgctacccagctgtatcagacttgcaaggcgaccggcacctgcccgcctgacgttatcccgaaagtggaacacactaccatcgcagaccagatcctgaaagcttccgcaacccagctgtacaaaacttgcaaacaggctggcacctgccctccggatatcattccgaaagtagaaggcaaaaccatcgccgaccagattctgcaggcaagcgtgactgatctgtacaaaacctgtaagcaatccggtacctgtccgccggacgtcgtgccgaaagtcgaaggtaccaccctggctgacaagatcctgcaggcgagcgcgacccagctgtaccagacctgtaaagcagcgggtacttgcccgagcgacgtaatcccgaaaatcgaacacaccactatcgcagaccagattctgcgcgcgtccgcaacccagctgtaccagacctgcaaagccacgggcacttgcccaccggacgtaattccgaaagttgaaggctccactattgccgatcagatcctgaaagcgagcgctacgcagctgtaccgtacctgcaaagccgccggtacttgtccgccggacgtcatcccaaaagtagagggtaacactgttgctgatcagatcctgaaagcatctgcgaccgacctgtaccgtacttgtaaacagagcggtacctgtccgccggatgttgttgacaaggttgaaggtaccactctggcggataaaatcctgcaagcgtctgcgaccgatctgtaccgtacttgcaaacagtccggcacctgtccgccggatgtgattaacaaagtcgaaggtacgacgctggcggataagatcctgcaggcttccgttacgcagctgtactctacctgcaaagcggcaggtacctgtccgccggacgtggtgaacaaagtagaaggtaccaccctggctgataaaattctgcaggcgtctgctacccagctgtaccaaacttgcaaagccagcggcacgtgcccgcctgatgttattccaaaagtagaaggcacgacgattgcggaccagctgctgaaagcgagcgctactcagctgtataagacctgcaaactgtctggcacttgtccagaggatgttgttaacaaaattgaacagaagacctgggcagacaaaatcctgcaagcttctgctactcagctgtatcagacctgtaaagcttctggtacctgtccgccggacgttatcccaaaagttgaaggtaccaccatcgccgaccagatcctgcgcgcatctgctaccgatctgtataaaacgtgcaaacaggccggcacttgcccgtctgacgttatcaacaaagtggagggtactaccctggcggataaaatcctgcaggcgtccgcaacccagctgtataaaacctgtaaacaggcaggtacttgcccgcctgacgtaattccgaaagttgagggttccaccatcgccgataatattctgaaataatagctcgagtctaga

**11-88x8Δ Sequence**

GGATCCATGGCTAAATTCGTTGCGGCTTGGACTCTGAAAGCCGCGGCTGCATCCGCAACCCAACTGTACCAGACTTGTAAACTGACGGGCACCTGCCCACCGGACGTTATCCCGAAAGTCGAGCATAATACCATCGCGGATCAGATCCTGAAAGGCACGGGTGGCCGTACGGGCTATGTTCCGCTGCAGACCTCCGCCAAACCGTCTATCACCTCTGGCCCGATGGCCAAACGTGCGAGCGCAACGCAGCTGTACAAAACCTGTAAACAGGCGGGCACGTGTCCACCAGACATCATTCCGAAAGTGGAAGGCAAAACCATCGCGGACCAGATCCTGCAAGGCACGGGCGGTCGCACTGGTTACATTCCGCTGGGTACTCGCCCACCGACCGCGACTGATACTCTGGCACCGCGCGCAAGCGTTACTGACCTGTACAAGACCTGCAAGCAGTCCGGTACTTGTCCGCCGGATGTGGTGCCGAAAGTCGAAGGCACGACCCTGGCGGATAAAATCCTGCAGGGCACTGGCGGTCGCACCGGCTATATCCCGCTGGGTGGCCGCTCTAACACCGTTGTGGACGTCGGTCCGACCCGCAAACGTGCGAGCGCGACCCAGCTGTATCAGACCTGCAAGGCGGCAGGTACCTGTCCGAGCGACGTGATCCCGAAAATCGAACACACTACGATTGCTGACCAGATTCTGCGTGGTACCGGTGGTCGTACGGGCTACGTCCCTCTGTCCACCCGCCCTTCTACTGTTTCCGAAGCGTCTATCCCGCGCGCCTCTGCTACTGATCTGTATCGTACCTGCAAACAGTCTGGCACTTGCCCGCCTGACGTTGTTGACAAGGTTGAAGGCACCACTCTGGCCGACAAAATCCTGCAGGGCACTGGTGGCCGTACTGGTTACATCCCACTGGGTGGTCGTCCTAACACCGTTGTTGACGTAAGCCCGGCTCGTCGTGCGAGCGTAACTCAGCTGTATTCCACTTGCAAGGCCGCTGGCACCTGCCCGCCGGATGTGGTTAACAAAGTAGAAGGTACGACCCTGGCAGATAAGATCCTGCAGGGTAGCGGCGGTCGTACTGGTTATATTCCACTGGGCGGTGGCGGTCGTCCGGGTGTGGTAGATATCGCACCGGCTCGTGCATCCGCGACCCAGCTGTACAAGACCTGTAAACTGAGCGGTACCTGCCCGGAAGACGTAGTGAACAAAATTGAGCAGAAAACCTGGGCTGATAAAATTCTGCAGGGTTCCGGCGGTCGTGCCGGTTACGTTCCGCTGGGCTCCCGCCCGAGCACCATCGTCGATGTAACCCCGGCTCGTAAAAAGCGTGCATCTGCAACCCAACTGTACAAAACCTGCAAACAAGCTGGTACCTGCCCTCCGGATGTAATCCCGAAAGTTGAGGGTTCTACCATTGCTGACAACATCCTGAAAGGTTCCGGTGGTCGTACCGGTTACGTGCCGCTGTCTACGGGTACCCCGTCTAAACCGGTGGAAATTCCATAGTAACTCGAG

**11-88x8ΔP Sequence**

GGATCCATGGCTTCCGCAACCCAACTGTACCAGACTTGTAAACTGACGGGCACCTGCCCACCGGACGTTATCCCGAAAGTCGAGCATAATACCATCGCGGATCAGATCCTGAAAGGCACGGGTGGCCGTACGGGCTATGTTCCGCTGCAGACCTCCGCCAAACCGTCTATCACCTCTGGCCCGATGGCCAAACGTGCGAGCGCAACGCAGCTGTACAAAACCTGTAAACAGGCGGGCACGTGTCCACCAGACATCATTCCGAAAGTGGAAGGCAAAACCATCGCGGACCAGATCCTGCAAGGCACGGGCGGTCGCACTGGTTACATTCCGCTGGGTACTCGCCCACCGACCGCGACTGATACTCTGGCACCGCGCGCAAGCGTTACTGACCTGTACAAGACCTGCAAGCAGTCCGGTACTTGTCCGCCGGATGTGGTGCCGAAAGTCGAAGGCACGACCCTGGCGGATAAAATCCTGCAGGGCACTGGCGGTCGCACCGGCTATATCCCGCTGGGTGGCCGCTCTAACACCGTTGTGGACGTCGGTCCGACCCGCAAACGTGCGAGCGCGACCCAGCTGTATCAGACCTGCAAGGCGGCAGGTACCTGTCCGAGCGACGTGATCCCGAAAATCGAACACACTACGATTGCTGACCAGATTCTGCGTGGTACCGGTGGTCGTACGGGCTACGTCCCTCTGTCCACCCGCCCTTCTACTGTTTCCGAAGCGTCTATCCCGCGCGCCTCTGCTACTGATCTGTATCGTACCTGCAAACAGTCTGGCACTTGCCCGCCTGACGTTGTTGACAAGGTTGAAGGCACCACTCTGGCCGACAAAATCCTGCAGGGCACTGGTGGCCGTACTGGTTACATCCCACTGGGTGGTCGTCCTAACACCGTTGTTGACGTAAGCCCGGCTCGTCGTGCGAGCGTAACTCAGCTGTATTCCACTTGCAAGGCCGCTGGCACCTGCCCGCCGGATGTGGTTAACAAAGTAGAAGGTACGACCCTGGCAGATAAGATCCTGCAGGGTAGCGGCGGTCGTACTGGTTATATTCCACTGGGCGGTGGCGGTCGTCCGGGTGTGGTAGATATCGCACCGGCTCGTGCATCCGCGACCCAGCTGTACAAGACCTGTAAACTGAGCGGTACCTGCCCGGAAGACGTAGTGAACAAAATTGAGCAGAAAACCTGGGCTGATAAAATTCTGCAGGGTTCCGGCGGTCGTGCCGGTTACGTTCCGCTGGGCTCCCGCCCGAGCACCATCGTCGATGTAACCCCGGCTCGTAAAAAGCGTGCATCTGCAACCCAACTGTACAAAACCTGCAAACAAGCTGGTACCTGCCCTCCGGATGTAATCCCGAAAGTTGAGGGTTCTACCATTGCTGACAACATCCTGAAAGGTTCCGGTGGTCGTACCGGTTACGTGCCGCTGTCTACGGGTACCCCGTCTAAACCGGTGGAAATTCCATAGTAACTCGAG
